# Sex Pheromones of the Potato Tuber Moth (*Phthorimaea operculella*)

**DOI:** 10.3389/fchem.2022.882400

**Published:** 2022-03-17

**Authors:** Huanhuan Pan, Hongyi Zhao, Likun Ai, Jian Huang, Yang Chen

**Affiliations:** State Key Laboratory Breeding Base of Green Pesticide and Agricultural Bioengineering, Key Laboratory of Green Pesticide and Agricultural Bioengineering, Ministry of Education, Research and Development Center for Fine Chemicals, Guizhou University, Guiyang, China

**Keywords:** sex pheromones, potato tuber moth (Phthorimaea operculella), synthesis, biological control, natural products

## Abstract

The potato tuber moth (*Phthorimaea operculella*) is a major potato pest. Its sex pheromones contain two chemical structures: 4E,7Z-tridecadiene-1-ol acetate (PTM1) and 4E,7Z,10Z-tridecatriene-1-ol acetate (PTM2). Increasing global consciousness of environmental protection is driving widespread attention to the possible use of these pheromones for sustainable pest management. This review summarizes research on the structure confirmation, field application, and chemical synthesis of the sex pheromones of the potato tuber moth. An efficient synthesis strategy of the two sex pheromones is proposed.

## Introduction

**FIGURE 1:**
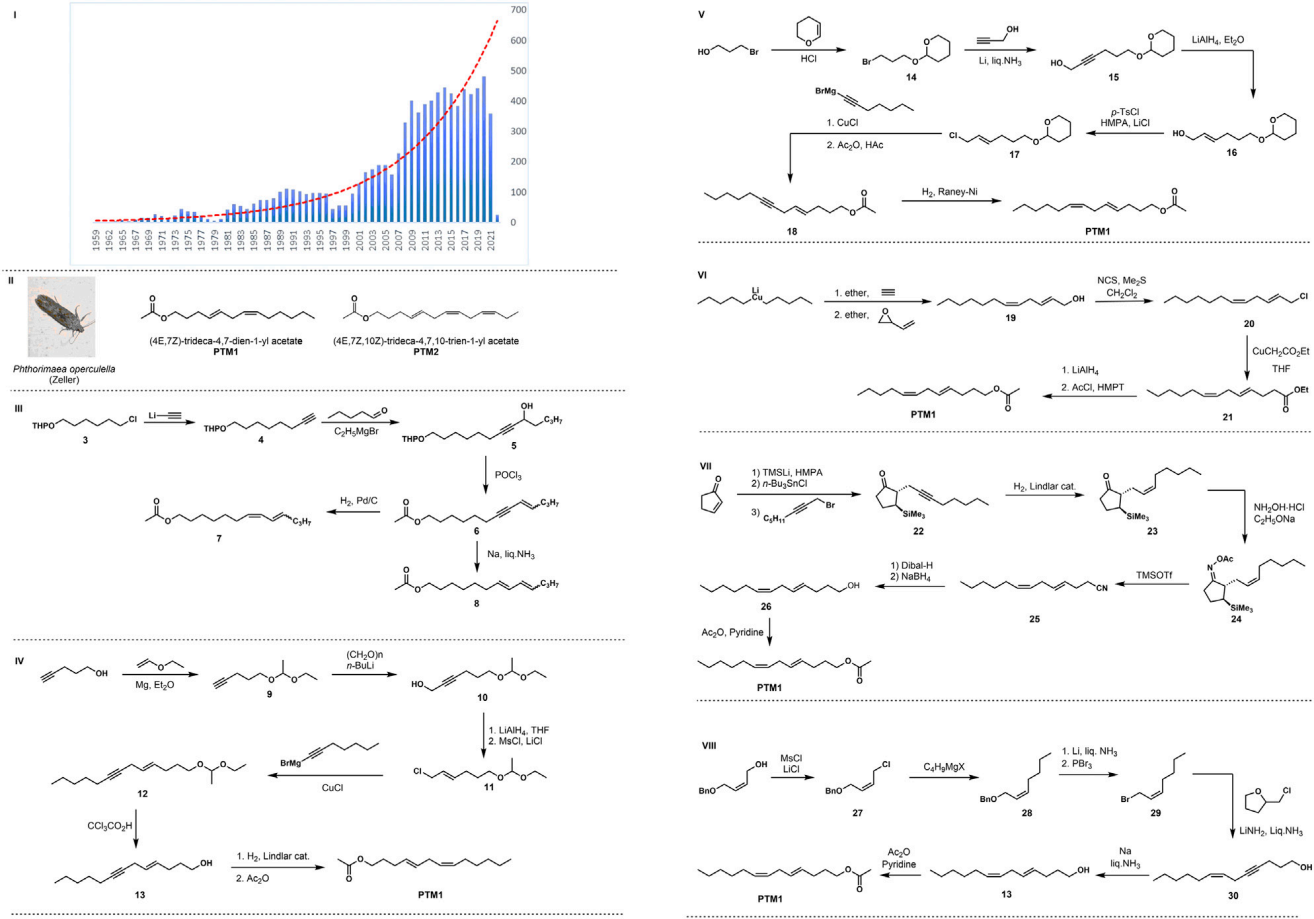
I Statistics of the number of research papers on insect sex pheromone from *web of science*. **II** Chemical structure of sex pheromone of potato tuber moth. **III** Synthesis of sex pheromone analogues of potato tuber moth by Fouda in 1975. **IV** Roelofs’ synthesis of PTM1 in 1975. **V** Voerman’s synthesis of PTM1 in 1978. **VI** Alexakis’s synthesis of PTM1 in 1978. VII Nishiyama’s synthesis of PTM1 in 1984. **VIII** Yadav’s synthesis of PTM1 in 1986.

Insect sex pheromones are used in environmentally friendly pest management. A large number of insect sex pheromones have been isolated and identified to control harmful insects in agriculture, horticulture, forestry, and stored products (Shen et al., 2020; Witzgall et al., 2010). Since the beginning of the 21st century, the attention of this field has increased exponentially, and the number of papers and the frequency of citations have increased yearly (Figure 1I). According to the statistics of *Web of Sciences in 2022* (Web of Scienece, 2022), the total number of papers on insect sex pheromones has reached 8,828, the H index has reached 146, and the cumulative citation frequency has reached 213,502 times.

Insect sex pheromones have some common characteristics: most of them are aliphatic long-chain olefin compounds that are volatile, they are safe for target organisms and non-target organisms because they have no direct killing effect, and they are mixtures of compounds in specific proportions that show strong specificity and high biological activity.

In China, potatoes play a key role in the national food security strategy and poverty alleviation strategies (Gao et al., 2019b). However, with the continuous expansion of potato planting areas, the occurrence of pests and diseases in production and storage is becoming increasingly severe (Xu et al., 2019). Traditional pests and diseases such as potato bacterial wilt, morning and late blight, and underground pests have been increasing year by year, as have outbreaks of insect pests such as the twenty-eight-star lady beetle (*Henosepilachna vigintioctopunctata*), potato tuber moth (*Phthorimaea operculella*), potato golden nematode (*Globodera rostochiensis*), potato beetle (*Leptinotarsa decemlineata*) and other pests. Changes in planting structure and scale together with global climate change further cause these hazards to spread. The potato tuber moth has developed into an important pest in China’s main potato producing areas such as Sichuan, Yunnan, and Guizhou.

The female potato tuber moth (also known as the tobacco leaf miner) lays multiple eggs individually on the ground and in soil crevices at the root of potato stems, near the midrib of the dorsal leaf, or between the petioles and axillary buds. For stored potatoes, adults prefer to lay eggs near the bud eye after the potato nubs are unearthed. In the field, the first-instar larvae start spinning silk after the eggs hatch, generally completing their larval stage in the leaves, and the old larvae fall into the soil to pupate. During the storage period, the larvae mostly enter from the bud eye or the cracked skin and gradually penetrate the potato pieces. After spinning silk the larvae form a worm tunnel (Coll and Gavish, 2000) and can consume much if not all of the potato flesh, causing the tubers to rot and lose their planting and edible value (Xie, 2014).

The early prevention and control of potato tuber moth mainly involve cultural control measures such as planting resistant varieties, deep seeding, and irrigation, but chemical control is still the main control method during potato production. Due to over-reliance on chemical pesticides, potato tuber moths have developed different degrees of resistance to pesticides such as organophosphorus and pyrethroids (Xu et al., 2019; Yan and Gao, 2019). With the gradual enhancement of people’s awareness of environmental protection, the research on insect sex pheromones has attracted extensive attention from agricultural biologists and chemists, as they provide a possibility to reduce dependence on chemical pesticides. This field is gradually becoming a hot spot in the field of sustainable plant protection (Wright, 1964).

## Structure Identification of the Potato Tuber Moth Sex Pheromone

Sexually mature female potato tuber moths were first described to use sex pheromones to attract males in the late 1960s (Adeesan et al., 1969; Ono et al., 1972). Compounds with strong attracting effect were first isolated from female potato tuber moths in 1975 (Fouda et al., 1975). Due to the limitations of structural analysis techniques at that time, only chemical tests, gas chromatography, and mass spectrometry were used to preliminarily determine the pheromone’s structure, which was thought to be isomers containing unsaturated double bonds and acetyl ester groups. In the same year, Roelofs (Roelofs et al., 1975) extracted and isolated a compound from the glands of the female potato tuber moth, and its structure (Figure 1II:4E,7Z-tridecadien-1-ol acetate (PTM1)) was determined by fine gas chromatography coupled with mass spectrometry, chemical synthesis, and bioactivity validation. One year later, Yamaoka et al. (1976) isolated the sex pheromones produced by potato tuber moth adults from unmated female moths raised in the laboratory. The results of gas chromatography and mass spectrometry showed that the pheromone was tridecatrienol acetate. They speculated that the pheromone structure was 4,7,10-Tridecatrienoate, but the configuration of its double bond had not been determined. In the same year, Persoons (Persoons et al., 1976) reported the extraction, isolation, structural identification, and field activity of the sex pheromone, and found that it was a mixture of two compounds as shown in Figure 1II. These compounds are 4E,7Z-tridecadien-1-ol acetate (PTM1) and 4E,7Z,10Z-tridecatrien-1-ol acetate (PTM2).

Voerman (Voerman et al., 1977) summarized a review on these sex pheromones and the two compounds are mixed in a certain proportion to exert the induction effect.

## Field Application of Potato Tuber Moth Sex Pheromone

Used in the field for more than 50 years, a variety of effective methods have been developed to measure the population size of potato tuber moths and control their damage to potato crops using sexual inducement technology.

Bacon (Bacon et al., 1976) used eight kinds of tridecene and tridecen-1-ol acetate monomers (including PTM1) or their mixtures to carry out chemical trapping experiments of potato tuber moths in the field, and found that PTM1 obtained the highest number of catches. Persoons (Persoons et al., 1976) found that the mixture of PTM1 and PTM2 had a synergistic effect in a field trapping experiment using mixing ratios from 4:1 to 1:4, but the use of any one of the monomers alone had almost no activity. In 1980, Ei-Garhy (1980) used a mixture of PTM1 and PTM2 sex pheromones smeared on a rubber lure core to capture male potato tuber moths in the field, and found that the environmental temperature and relative humidity had a very significant effect on trapping efficiency. Raman (1982); Raman (1984); Raman (1988) conducted a large number of field trapping activity experiments using PTM1 and PTM2, and found that the mixture of PTM1 and PTM2 was more attractive than PTM1 alone. In 1982, the research group used a mixture of PTM1 and PTM2 in eight different ratio formulations to conduct capture tests and found that the highest capture rate was initially obtained when the ratio of PTM1 to PTM2 was 9:1, 3:1, or 1:1.5. However, after 90 days in the field, the attractiveness of the 9:1 ratio mixture decreased, while the 1:1.5 ratio remained attractive after 90 days. Storage at −5°C for 2 months did not reduce the attractiveness of the mixture. Toth (Toth et al., 1984) studied the response of male potato tuber moths to two sex pheromone components and female crude extracts and found that in wind tunnels and fields, compared with compounds composed of trienes alone, males were better able to localize by a mixture of PTM1 and PTM2 (1:1). The addition of PTM1 reduced the time male moths spent near the pheromone source and the pheromone itself compared to PTM2 alone, also increasing the average number of visits to the source; and male potato tuber moths were also found to have behavioral responses to a mixture of PTM1 and PTM2 (1:1) similar to those elicited by the female crude extract. From 1972 to 2016, Ono (1993); Ono (1994); Ono et al. (1997); Ono and Orita (1986) and Tejima et al. (2013); Tejima et al. (2016) carried out systematic research on the structure and properties of potato tuber moth sex pheromones. In 1986, they found that the ratio of the sex pheromones was affected by temperature. As the feeding temperature increased, PTM2 gradually decreased, but the total amount of sex pheromones did not change. The pupal stage was the most sensitive to temperature changes. Kroschel and Zegarra (Kroschel and Zegarra, 2010; Kroschel and Zegarra, 2013) studied the use of the potato tuber moth pheromones and a single structural pheromone of *Symmetrischema tangolias* combined with the insecticide cyfluthrin to form an attract-kill system, which resulted in 100% mortality in males under laboratory conditions. In field and storage conditions, the trapping method was very effective against potato tuber moths and represents a low-cost control method inducible under storage conditions that can be effectively integrated into potato pest control programs, especially in tropical and subtropical small farming systems. From 2018 to 2019, Gao et al. (2018); Gao et al. (2019a) developed a “comprehensive green control technology for potato tuber moths based on sexual attractants”, which integrates a number of prevention and control measures for different stages of potato tuber moth. The application of sexual attractant technology interferes with the normal mating of female adults in the field, and the lack of mating leads to an increase in unviable eggs. After the technology was applied in Qujing, Yunnan, for one to 2 years, the number of potato tuber moths was greatly reduced, and the damage was alleviated.

## Synthesis of Potato Tuber Moth Sex Pheromone by Predecessors

So far, the source of potato tuber moth sex pheromone mainly relies on extraction from female insects. Due to the low content in the insect source glands, the separation efficiency is not high, and this source cannot meet the needs of a wide range of field applications. Since the structures of the potato tuber moth sex pheromones (PTM1 and PTM2) were first reported, their structural specificity and remarkable sex-inducing activity have attracted many chemists to attempt the chemical synthesis of these two molecules.

As shown in Figure 1III, Fouda et al. (1975) used commercially available tetrahydropyran-protected 6-chloro-1-hexanol as the starting material, and then reacted it with ethynyl lithium to generate compound 4, which was coupled with n-hexene to obtain intermediate 5. Then the elimination reaction took place under the action of phosphorus oxychloride to obtain intermediate 6. The target product 7 (7Z, 9E/Z-tridec-dien-1-ol acetate) was then obtained by catalytic hydrogenation with Pd/C, and the alkyne bond was reduced in parallel with liquid sodium ammonia to obtain the target compound 8 of E-configured olefin (7E, 9E/Z-tridec-dien-1-ol acetate).

In the same year, Roelofs et al. (1975) started their synthesis with a coupling reaction between 4-pentyn-1-ol and vinyl ethyl ether to obtain acetal 9, and propynyl alcohol 10 was gained by reaction with paraformaldehyde and *n*-BuLi. Followed by reduction with LiAlH_4_, hydroxychlorination under the combined action of MsCl/LiCl/2,4,6-trimethylpyridine to obtain Chloride 11, as illustrated in Figure 1IV. Coupling with alkynyl Grignard reagent under the action of CuCl gave compound 12, the protecting group is subsequently removed and alkyne bond was reduced by Lindlar catalyzed stereoselective hydrogenation to obtain Z-configuration double bond. Finally, the terminal primary alcohol hydroxyl group is acetylated to obtain the target compound PTM1.

In 1978, Voerman and Rothschild (1978) used 3-bromo-1-propanol as the starting material, first protected the alcoholic hydroxyl group under the action of dihydropyran to obtain intermediate 14, and then reduced it with propargyl alcohol by Birch to generate alkyne alcohol 15. Then, with *p*-toluenesulfonyl chloride as the halogen source and *n*-butyllithium as the strong base, hydroxychlorination occured to obtain the alkene halogen compound 17, which was then coupled with the alkynyl Grignard reagent to remove the protective group with cuprous chloride to obtain compound 18, as shown in Figure 1V. Finally, the target compound PTM1 was obtained by hydroxyacetylation and Raney nickel-catalyzed hydrogenation to reduce the alkynyl group.

The same year, Alexakis et al. (1978) reported the total synthesis route of PTM1. The key step is the ring-opening coupling reaction between an organocopper reagent and an allyl epoxy compound, as illustrated in Figure 1VI. This route uses lithium dipentyl ketone as the starting material. First, the key E, Z diene compound 19 is prepared by addition coupling and conjugated ring opening, and then the intermediate 21 is obtained by hydroxy chlorination and alkylation. This is followed by ester reduction and hydroxyacetylation to give the pheromone PTM1.

In 1984, Nishiyama et al. (1984) used the silicon-guided Beckmann fragmentation strategy to develop several new methods for the stereo-controlled synthesis of insect sex pheromones. The key is to construct the E-type double bond through the trimethylsilyl auxiliary region and the stereo-controlled Beckmann rearrangement reaction. This route uses 2-cyclopentenone as the starting material, reacts with trimethylsilyllithium and n-tributyltin hydride in turn, and then reacts with octynyl bromide to obtain compound 22, which is hydrogenated by Lindlar catalyzed to obtain Z-configuration alkene 23, as shown in Figure 1VII. Subsequently, it was reacted with hydroxylamine hydrochloride to form oxime acyl ester 24, and then the diene cyano compound 25 was obtained by silicon-promoted Beckmann fragmentation reaction under the action of TMSOTf silicon reagent, and finally potato Tuber moth sex pheromone PTM1 was obtained through two reductions and hydroxyacetylation.

In 1986, Yadav and Reddy (1986), using Z-alkene-1-ol as the starting material, first obtained Z-form alkene chloride 27 by hydroxychlorination, and then reacted with Grignard reagent for carbon chain growth and coupling reaction to obtain alkene benzyl ether 28, as illustrated in Figure 1VIII. Subsequent Birch reduction to remove the benzyl group and hydroxy bromination in two steps produced intermediate 29, which was reacted with 4-pentyn-1-ol prepared with tetrahydrofuroyl chloride through a diionic reaction to obtain primary alkenyl alkyne primary alcohol 30. Finally, Birch reduction of the alkynyl group and primary alcohol hydroxyacetylation completed the total synthesis of PTM1.

**FIGURE 2:**
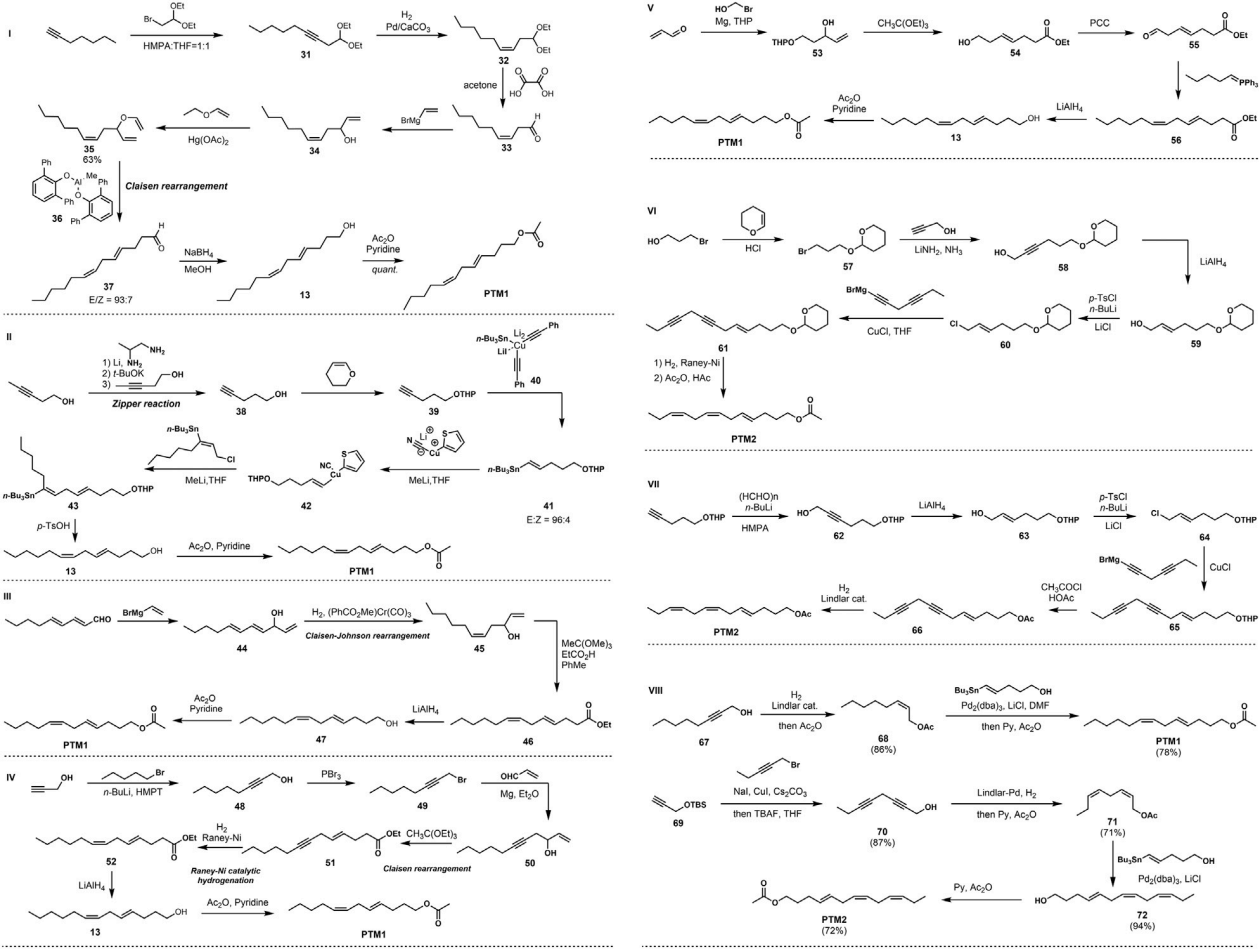
I Nonashita’s synthesis of PTM1 via Claisen rearrangement in 1990. II Hutzinger’s synthesis of PTM1 in1995. III Vasil’ev’s synthesis of PTM1 in 1996. IV Odiokov’s synthesis of PTM1 in 1997. V Vakhidov’s synthesis of PTM1 in 2007. VI Voerman’s synthesis of PTM2 in 1978. VII Fukami’s synthesis of PTM2 in 1978. VIII Our group’s synthesis of PTM1 and PTM2 via Stille coupling in 2021.

In 1990, Nonoshita et al. (1990) developed the use of bulky, sterically hindered diarylmethylaluminum as a key reducing agent for Claisen rearrangement to construct E-form double bonds with high selectivity, as shown in Figure 2I. Using 1-heptyne as the starting material, the rearranged precursor allyl vinyl ether 31 was obtained through SN2 reaction, Lindlar reduction, deacetalization, and Grignard reagent coupling and substitution. The Claisen rearrangement reaction occurred under the action of large sterically hindered diarylmethylaluminum 37, and the diene aldehyde with the E configuration was obtained with high stereoselectivity. Finally, the target product PTM1 was obtained through two-step conversion of aldehyde group reduction and primary alcohol acetylation.

In 1995, Hutzinger and Oehlschlager (1995) developed a method for the stereoselective synthesis of Z, E- and 1Z, 4Z-dienes by cross-coupling of allyl substrates with vinyl organometallic reagents, and used this method to synthesize PTM1. The synthetic route uses 3-pentyn-1-ol as the starting material, obtains 4-pentyn-1-ol through the Zipper reaction, and then protects the hydroxyl group to obtain compound 39. The E-configuration olefin 41 was obtained under the action of an organocopper reagent, as illustrated in Figure 2II. This is then coupled with allyl chloride under the action of lithium 2-thiophene cyanoate to obtain diolefin 42, and finally the hydroxy THP protecting group is removed and acetylated to obtain the target compound PTM1.

One year later, Vasil’ev and Serebryakov (1996) used 2E, 4E-nonadienal and vinylmagnesium bromide as starting materials, and obtained triene secondary alcohol 33 through the Grignard reaction, and then 1,4-selective reduction of conjugated olefins took place under the action of the aromatic chromium tricarbonyl to generate Z-type olefins 45, as illustrated in Figure 2III. Subsequently, Claisen-Johnson rearrangement occurred in the presence of trimethyl orthoacetate and a small amount of propionic acid to obtain (4E, 7Z)-configured diene 46, and finally ester reduction and hydroxyacetylation occurred to obtain the target compound PTM1.

In 1997, Odinokov et al. (1997) started their synthesis with a coupling reaction between propargyl alcohol and 1-bromopentane to build alkynol compounds. Then, under the action of phosphorus tribromide, hydroxy bromination occured to obtain propargyl bromide, which was then prepared into propargyl magnesium bromide Grignard reagent, and a Grignard addition reaction occured with acrolein to obtain the key intermediate 50, as illustrated in Figure 2IV. Subsequent intramolecular Claisen rearrangement under the action of triethyl orthoate generated compound 51. Finally, through Raney-Ni catalyzed hydrogenation reduction to 7Z double bond, ester reduction to alcohol, and hydroxyacetylation, two simple transformations achieved the total synthesis of the target PTM1.

In 2007, Vakhidov and Musina (2007) reported a new method for the synthesis of PTM1 using the Claisen rearrangement reaction and the Wittig reaction, as shown in Figure 2V. Using acrolein and 2-bromoethanol as starting materials, hydroxyaldehyde 53 was first prepared by Grignard reaction, and then intramolecular Claisen rearrangement occurred in the presence of triethyl orthoacetate as THP was simultaneously removed to generate E-form olefin carboxylate 54, followed by PCC oxidation and Wittig reaction to give 55, and finally ester reduction and hydroxyacetylation to achieve the synthesis of PTM1.

There are relatively few synthetic examples of PTM2, with only two cases reported. In 1978, Voerman and Rothschild (1978) completed the synthesis PTM2 adopting the same strategy as shown in Figure 2VI. After allyl chloride 60 is prepared, it is coupled with diacetylene under the catalysis of cuprous chloride to obtain 4E-7,10-diacetylene 61, followed by Raney-Ni catalytic hydrogenation to obtain 4E-7Z,10Z-trien-1-ol, and finally acetylation to obtain the target compound PTM2.

That same year, Fukami et al. (1978) used dihydropyran-protected as the starting material, and obtained allyl chloride 64 through chain extension, acetylenic bond reduction, and hydroxyl halogenation, as shown in Figure 2VII. This was coupled with a dialkynyl Grignard reagent to obtain 4E-7,10-dialkyne 65, followed by replacement of the hydroxyl protecting group, and finally the synthesis of the potato tuber moth sex pheromone PTM2 by Lindlar hydrogenation reduction.

## Synthesis of Potato Tuber Moth Sex Pheromone by Our Group

The difficulty and challenge for PTM1 and PTM2 lie in the high stereoselectivity construction of the double bonds. In the early reports of sporadic synthesis methods, the construction of E and Z double bonds mainly included Grignard reagent coupling, Wittig reaction, etc., but these methods have the defects of low yield and vague selectivity. Based on this, our research group has carried out research on the synthesis of the potato tuber moth sex pheromones PTM1 and PTM2, as shown in Figure 2VIII. The goal was to use Lindlar-catalyzed hydrogenation and Stille coupling as key reactions to realize the construction of Z and E double bonds with high selectivity and yield.

This scheme uses commercial tert-butyldimethyl (2-propynyloxy) silane as the starting material, first reacting it with 1-bromo-2-pentyne and removing the TBS protecting group to give the bisalkynyl compound 70 by TBAF. At the same time, using 2-octyn-1-ol as the starting material, the key intermediates 67 and 70 were obtained by the catalytic hydrogenation of Lindlar and the protection of the primary alcohol by the acetyl group. Subsequently, the construction of the E-type double bond was achieved by Stille coupling under the action of tris(dibenzylideneacetone)dipalladium. Finally, the primary alcohol was acetylated to obtain the target compounds PTM1 and PTM2, with the overall yields of 67 and 42% (Gao et al., 2018).

## Summary

Potatoes are harmed by various pests such as potato tuber moth, and the average yield loss can reach 40–45%. However, the acquisition of the sex pheromone of potato tuber moth is mainly obtained from the glands of female moths. This method is inefficient and not enough to support the application of field experiments. The new method developed by our group has low cost, and has important scientific significance and application prospect for realizing the technological production of potato tuber moth sex pheromone.
